# Improving reflective evaluations of sport through repeated experiences of fun—rationale, design, feasibility, and acceptability of the PlayFit Youth Sport Program

**DOI:** 10.1186/s40814-023-01350-x

**Published:** 2023-07-10

**Authors:** Matthew A. Ladwig, Christopher N. Sciamanna, Gavin Luzier, Joshua M. Blaker, Jennifer P. Agans, Amanda J. Visek

**Affiliations:** 1 Purdue University Northwest, Hammond, IN USA; 2 Penn State College of Medicine, Hershey, PA USA; 3 Department of Biological Sciences and Integrative Physiology and Health Sciences Center, Purdue University Northwest, Hammond, IN 46323 USA; 4 Penn State University, University Park, PA USA; 5 The George Washington University, Washington, DC USA

**Keywords:** Positive youth development, Affect, Emotion, Sport attrition, Adherence, Motivation, Intervention development

## Abstract

**Introduction:**

Adolescents who drop out of sport often report that it had become less ‘fun’ and ‘enjoyable’ over time. Although preadolescent sport typically emphasizes experiences of fun, emphases on competition and elite performance often dominate during adolescence. We theorized that adherence to adolescent sport might be improved if the overarching goal were to maximize repeated experiences of fun during sport and, subsequently, increase reflective evaluations of sport enjoyment. To that end, this manuscript reports on the rationale and design of the PlayFit Youth Sport Program (PYSP), as well as its preliminary feasibility and acceptability. The main objectives were to evaluate the feasibility of recruitment strategies and data collection procedures and the acceptability of the intervention.

**Setting:**

An outdoor, multipurpose grass field at a south-central Pennsylvania middle school.

**Methods:**

A mixed-methods, single-arm feasibility trial lasting for 8 weeks (August–October 2021) offered 3-times per week for 1-h per session. The equipment, ruleset, and psychosocial environment of the PYSP sport games were modified to reduce several of the constraints theorized to impair experiences of fun during sport and hamper reflective evaluations of enjoyment afterward.

**Results:**

Eleven healthy, but sedentary adolescents in grades 5–7 completed the program. The median number of sessions attended (of 16 possible) was 12 (range = 6–13). Post-intervention, 9/10 respondents indicated that they ‘looked forward’ to the PYSP, 8/10 would recommend it to a friend, and 8/10 were interested in continuing the program. Ten of 11 participant guardians expressed interest in reenrolling their children if the PYSP were offered again. Some changes recommended were to improve recruitment via advertising the positive aspects of the program and “word of mouth” techniques, offering the program immediately following the school day, having contingencies for inclement weather, and minor changes to the sport equipment to improve the experience among the population the PYSP intends to attract.

**Conclusions:**

The adjustments recommended in this preliminary work could be used to further refine the PYSP. A future efficacy trial could explore whether the PYSP may reduce attrition for adolescents who experience existing sport programs negatively by offering an alternative that better matches their unique needs and preferences.

**Supplementary Information:**

The online version contains supplementary material available at 10.1186/s40814-023-01350-x.

## Key messages regarding feasibility

• What uncertainties existed regarding feasibility?

- ◦ We were unsure whether sedentary adolescents would express interest, enroll, and adhere to the PlayFit Youth Sport Program (PYSP). In addition, we wondered whether the time of day, duration, and frequency of sessions were ideal for participant recruitment and retention.

• What are the key feasibility findings?

- ◦ Ninety-two percent of adolescents who enrolled completed the PYSP intervention and 80% expressed interest in continuing to participate. Additionally, we learned that offering sessions earlier in the day (e.g., following school) and having contingencies in place for inclement weather (i.e., ability to move indoors), could increase the number of sessions available and daily attendance. The participants also offered suggestions to improve the experience via minor changes to the sport equipment of the games.

• What are the implications of the feasibility findings for the design of the main study?

- ◦ The PYSP intervention could be a promising approach to physical activity promotion among sedentary adolescents, but alternative advertising strategies may be required to increase recruitment. Prior to conducting an efficacy trial of the PYSP, the recommendations for improvement gathered from the present study should be considered.

## Introduction

Recent estimates suggest that approximately one-third of youth sport participants drop out annually and 70% of all children will have done so by 13 years of age [1–3]. Moreover, up to 80% of adolescents are insufficiently active [4–9], suggesting that those who drop out of sport may not be substituting physical activity (PA) through sportwith participation in alternative physical activities. These inactive children may be less likely to realize the many physical and mental health benefits [10–15] of regular PA. Inactivity during childhood also tends to track into adulthood [16–18] ﻿—meaning it could become intergenerational﻿—as adults often influence the PA behavior of their children [19, 20]. Despite these concerns, interventions that reduce, and, perhaps, reverse youth sport attrition have been characterized as ‘urgently needed’ since at least the 1980s [21–23].

There are numerous explanations for adolescent sport attrition (e.g., lack of time, competing priorities, poor access, high costs; [24–27], but one deserving more attention could be that those who drop out of sport frequently report that it was no longer fun and enjoyable [28–31]. While most youth athlete development models [32, 33] suggest that childhood sport should maximize experiences of fun and enjoyment, adolescent sport programming often focuses on elite performance, competitive success, and progressing athletes to the ‘next level’ [22, 23, 33, 34]. ‘Having fun’ during sport may be incompatible with these outcomes [35], as those who play to have fun are seen as ‘carefree’ [36] 'goofing off,' or 'not caring' about the outcome [37]. Perhaps due to this negative connotation, the term 'fun' appears frequently in the earlier (i.e., younger) stages of the Developmental Model of Sport Participation [32] and Long-Term Athlete Development Model [33] but receives little attention in later stages. This shift toward focusing on performance and competition outcomes could introduce several of the intrapersonal, interpersonal, and structural constraints that are theorized to reduce experiences of fun [30].

Ideally, so-called recreational sport would provide an alternative to elite and competitive sport for adolescents who wish to participate primarily to have fun. Indeed, the explicit missions of many recreational sport organizations are to provide programming that balances fun, enjoyment, performance, and competition [38, 39]. At the same time, ‘recreational’ is a nebulous term, meaning what characterizes recreational sport can vary depending on the organization and its administrators. Moreover, the personalities, expertise, and training of the (often) volunteer coaches influence the participant experience [38, 40–42]. That is, recreational sport leaders are often current or former athletes who are well-versed in sport knowledge, but may have little coaching experience or expertise in positive youth development and motivation [38]. Consequently, the experience of recreational sport programs may closely resemble more ‘traditional’ competitive and elite programs—leaving many adolescents without programming to meet their unique needs and preferences. We suggest that, to mitigate youth sport attrition, an alternative pathway must be developed for those who negatively experience existing youth sport programming. The PlayFit Youth Sport Program (PYSP) could serve as one such alternative.

### Rationale for the PlayFit Youth Sport Program

Dual-process theories [43] guided the development of the PYSP. Dual process theories suggest that decision making is underpinned by the interactions between automatic associations and reflective (i.e., cognitive) evaluations. Accordingly, we suggest that sport becomes automatically associated with 'fun' when a player repeatedly and consistently experiences pleasure and positive emotions *during* participation. *Afterward*, the player more slowly and deliberatively reflects on the quality of their experiences during sport. A player may, for example, consider whether the experience fostered perceptions of competence, autonomy, and relatedness [44]. These retrospective evaluations coalesce to form an overall positive or negative reflective evaluation of sport. Critically, dual-process theories suggest that behavior is more likely when automatic associations and reflective evaluations are concordant [45]. That is, a player who both automatically associates sport with fun and has a positive reflective evaluation of it is more likely to participate than someone who has high perceived sport competence but does not automatically associate sport with being fun. Consequently, the philosophy of the PYSP is that repeated and consistent experiences of fun should be the ‘prime directive’ (i.e., principal emphasis) that supersedes all others (e.g., changes in fitness, skillfulness, performance).

The PYSP leverages several theoretical models and empirical findings to help mitigate the myriad constraints [30] that may reduce experiences of fun during and degrade reflective evaluations after sport participation. For instance, fun-integration theory (FIT; [46] posits that three ‘fun-dimensions’ (i.e., ‘trying hard,’ ‘positive team dynamics,’ and ‘positive coaching’) are important influences on fun during sport. Moreover, sport programming that fulfills the basic psychological needs of competence, autonomy, and relatedness [44] may lead to increased positive emotional experiences, stemming from perceptions of competent performances or playing with friends. In addition, experiences of fun during sport may be greater if the program culture matches the goal orientations [47] of the participants. That is, those with task (or mastery) goal-orientations may have less fun during sport if the culture encourages interpersonal comparisons of ability and performance instead of perceptions of personal achievement.

Findings from exercise psychology could also help augment the experience of fun during sport. For example, there is considerable interindividual variability in the affective responses (pleasure-displeasure) elicited by differing exercise intensities. Specifically, some people report feeling better, and others worse, during vigorous-intensity exercise. [48]. Moreover, many children and younger adolescents report decreasing pleasure throughout continuous and incremental exercise [49, 50]. Allowing these individuals to self-regulate their physical effort (i.e., slow down or stop if experiencing discomfort/displeasure) without negative consequences (e.g., being punished or shamed) may increase their experiences of fun during sport and improve their subsequent reflective evaluations. Finally, evidence from youth PA patterning [51, 52] suggests that children tend to gravitate toward movements that closely approximate the gameplay of 'tag' (i.e., intermittent light-to moderate-intensity PA, with shorter bursts of vigorous-intensity activity interspersed throughout). Sport programming that mirrors these seemingly inherent movement propensities may be more likely to be experienced as fun by children. Table 1 outlines in more detail how and where previous these theoretical models and empirical findings were incorporated into the design of the PYSP.

**Table 1:** Description of how the PlayFit Youth Sport Program addresses intrapersonal, interpersonal, and structural barriers to experiences of fun during sport and reflective evaluations of sport enjoyment

	How the PlayFit program addresses the constraint	Tenets of theoretical models incorporated
Intrapersonal constraints		
Perceptions of physical incompetence/Emphasis on high performance	• No ‘normative’ skill and experience level expectations to participate • Players are encouraged to positively reframe their own mistakes and tolerate those of others • Few skill and fitness barriers to entry (e.g., easier to use equipment, basic, easy to understand rules that allow for autonomy/creativity)	• Trying hard, positive team dynamics (fun-integration theory; FIT) • Task-orientation (achievement-goal theory; AGT) • Competence, autonomy, relatedness (self-determination theory; SDT)
Negative perceptions of coach/leader	• As actively participating teammates, leaders model the program ethos by positively reframing and tolerating mistakes • Leaders monitor the team dynamics to discourage antisocial behaviors (e.g., bragging, bullying, teasing, formation of cliques) • Leaders prevent participants from being excluded (e.g., by passing to players not receiving attention or ‘touches’) • Leaders value and praise prosocial behaviors (e.g., players who encourage one another, pass to teammates who do not frequently receive one, allowing another player to have a 're-do') and trying hard • Leaders develop supportive connections/relationships with participants (e.g., get to know on first name basis, interests beyond the program)	• Trying hard, positive coaching, positive team dynamics (FIT) • Task-orientation (AGT) • Competence, relatedness (SDT)
Negative perceptions toward teammates	• Participants agree to abide by program rules prior to participation, whereby failure to do so may result in dismissal • Players are encouraged to perform altruistic behaviors (e.g., providing praise and positive feedback, involving less skilled peers) • Players are encouraged to tolerate the mistakes of others • Players are discouraged from bragging, bullying, teasing, and the formation of cliques	• Trying hard, positive team dynamics (FIT) • Task-orientation (AGT) • Relatedness (SDT)
Interpersonal constraints		
Pressures from family, coaches, peers Other social priorities	• Parents/guardians are *not* encouraged to attend—if they must do so, they agree to observe sessions from afar • Program is coed and loosely age-restricted (e.g., 'all middle school children') so participants may socialize with similar peers • No obligation to adhere to program (i.e., will not ‘let team down’ if individuals decide to not attend) • Participants provided with more ownership of their experience and can 'come and go' as they please during sessions	• Autonomy, relatedness (SDT)
Structural constraints		
Overemphasis on competitive success	• No scorekeeping, standings, or statistics	• Trying hard, positive team dynamics, mental bonuses^a^ (FIT) • Task-orientation (AGT) • Competence, relatedness (SDT)
Time commitments	• No mandatory practices/games, attend and play as much or as little as desired • No obligation to adhere to the program (i.e., will not 'let team down' if they decide not to attend)	• Autonomy (SDT)
Not being given adequate playing time	• No ‘try-outs’ or ‘cutting’ from program – all who attend up may participate as much or as little as desired • Teams are randomly chosen daily	• Positive team dynamics, positive coaching (FIT) • Autonomy, relatedness (SDT)
Injuries, psychological, and physiological burnout	• Activities are non-contact • Participants encouraged to self-regulate effort to reduce chances of overuse injuries and feelings of displeasure from overexertion (i.e., slow down/take breaks/switch-out whenever desired) • Multisport and games change weekly • Non-competitive, low stress ethos to reduce likelihood of psychological burnout	• Positive team dynamics (FIT) • Supporting the often-intermittent patterning of youth physical activity • Allows for self-regulation of physical effort

^a^For a more detailed summary of the 11 dimensions and 81 individual fun-determinants postulated by fun-integration theory, readers are directed to Visek and colleagues (2015)

### Study objectives

The present study was designed to explore the preliminary feasibility and acceptability of the PYSP program to understand whether a future efficacy trial may be warranted. Because the terms *pilot* and *feasibility* are often used interchangeably, distinguishing between them can be difficult. For instance, Whitehead and colleagues [53] suggested that, in contrast to feasibility studies, pilot studies have stricter study methodology, an explicit intention for future work, are smaller versions of the primary trials (i.e., they often include randomization to groups), and focus on trial outcomes. We considered the present work a feasibility study as it was “iterative, formative, and adaptive [54]” and used flexible methodology that allowed changes to be made when necessary to improve intervention delivery [55]. To investigate the *feasibility* (i.e., the extent to which procedures are successfully delivered in a distinctive context that is not fully controlled) and *acceptability* (i.e., the extent to which procedures are perceived as fit, satisfying, and appealing) of the PYSP, we adapted the social and behavioral feasibility study data collection and evaluation framework suggested by Orsmond and colleagues [54] that included the following criteria (see Table 2 for more details):

1. Recruitment capability and resulting sample characteristics.
2. Evaluation and refinement of data collection procedures and outcome.
3. Acceptability and suitability of intervention and study procedures.
4. Resources and ability to manage and implement the study and intervention.
5. Preliminary evaluation of participant responses to intervention.

**Table 2:** Social and behavioral feasibility study design criteria suggested by Orsmond and colleagues [55]

Objective 1: Recruitment capability and resulting sample characteristics Main question: *Can we recruit appropriate participants?*
1. How many potential eligible members of the targeted population are accessible in the local community?
2. What are the recruitment rates?
a. How many participants enter the study at a time?
b. How long does it take to recruit enough participants into the study?
c. What are the refusal rates for participation?
3. How feasible and suitable are eligibility criteria?
a. Are criteria clear and sufficient or too inclusive or restrictive?
4. What are the obstacles to recruitment?
a. Are colleagues and local organizations willing to assist with recruitment?
b. What are the reasons for refusal or ineligibility?
5. How relevant is the intervention to the intended population?
a. Do study participants show evidence of need for the intervention?
6. Are the characteristics of the study participants consistent with the range of expected characteristics as informed by the research literature?
Objective 2: Evaluation and refinement of data collection procedures and outcome measures Main question: *How appropriate are the data collection procedures and outcome measures for the intended population and purpose of the study?*
1. How feasible and suitable are the data collection procedures?
a. Do participants understand the questions and other data collection procedures?
b. Do they respond with missing or unusable data?
2. How feasible and suitable is the amount of data collection?
a. Do the participants have the capacity to complete the data collection procedures?
b. Does the overall data collection plan involve a reasonable amount of time or does it create a burden for the participants?
3. Do the measures appear to be performing in a consistent way with the intended population as compared to measurement information available in the research literature?
a. Are internal consistency indicators of measures with the recruited sample congruent with expectations based on prior studies reported in the research literature?
b. Do planned outcome measures appear to be sensitive to the effects of the intervention?
c. Does a suitable outcome measure need to be developed?
Objective 3: Acceptability and suitability of intervention and study procedures Main question: *Are study procedures and intervention suitable for and acceptable to participants?*
1. What are the retention and follow-up rates as the participants move through the study and intervention?
2. What are the adherence rates to study procedures, intervention attendance, and engagement?
a. Does the intervention fit with the daily life activities of study participants?
b. Do the participants have enough time and capacity to complete the intervention?
c. Does the intervention involve a reasonable amount of time, or does it create a burden for the participants?
d. To what extent is the intervention acceptable and appealing to participants?
e. If appropriate, how many participants agree to be randomized to group?
3. What is the level of safety of the procedures in the intervention?
a. Are there any unexpected adverse events?
Objective 4: Resources and ability to manage and implement the study and intervention Main question: *Does the research team have the resources and ability to manage the study and intervention?*
1. Does the research team have the administrative capacity, expertise, skills, space, and time to conduct the study and intervention?
2. Can we conduct the study procedures and intervention in an ethical manner?
a. To what extent does staff comply with the approved human participants’ protocol?
b. How effectively are adverse events during implementation identified, documented, and reported?
3. Can the study and intervention be conducted within the designated budget?
4. Is the technology and equipment sufficient to conduct the study and intervention, including collection, management, and analysis of data?
a. Is equipment available when needed?
b. What is involved in training personal and/or participants to use the equipment?
5. Are we able to efficiently and effectively manage data entry and analysis?
Objective 5: Preliminary evaluation of participant responses to intervention Main question**:** *Does the intervention show promise of being successful with the intended population?*
1. Does examination of quantitative data suggest that the intervention is likely to be successful?
a. Does examination of the data at the participant level suggest that changes in key outcome variables occurred?
b. Are the changes of the outcome variable(s) in the expected direction?
c. Do the estimates of effects suggest that the intervention has promise?
2. Do participants or relevant others provide qualitative feedback that may be indicative of the likelihood that the intervention will be successful?
3. If the quantitative and/or qualitative data suggest that the intervention is not promising:
a. Are the data collection procedures and outcome measures appropriate for the population and study?
b. Are the outcome measures and intervention theoretically aligned?
c. Is there evidence that the intervention does not produce change in the desired outcomes?
d. Is there evidence that the intervention was not implemented in the intended manner?
e. Have too many adaptations been made in the intervention process to adequately assess the participants’ responses to the intervention?
4. Are the findings congruent with the proposed theoretical model for the intervention?

## Method

### Trial design and setting

A mixed-methods single-arm feasibility trial describing a *formative* version of the PYSP. All study activities took place in south-central Pennsylvania in the midst of the COVID-19 pandemic. As recommended by the editorial board for non-randomized feasibility studies focused on intervention development [56], our findings are reported using the applicable items from the Consolidated Standards of Reporting Trials (CONSORT)—extension to randomized pilot and feasibility trials [57] (see Supplementary files for CONSORT diagram). Human-subjects approval for the study was provided by the Institutional Review Board.

### Participants

We aimed to recruit up to 20 participants for this study. Males or females between the ages of 10 and 12 years of age who were healthy enough for physical activity [58], but sedentary (defined as participating in fewer than 60-min of moderate-to vigorous-intensity physical activity; [MVPA] per day) [59] were included. Individuals were excluded if they were unable to speak and read English, could not assent both verbally and in writing, and/or those whose guardians did not provide written informed consent. The participants were compensated for answering questionnaires at baseline and week 8.

### Intervention delivery

The local public school district provided a multi-use field to host the PYSP sessions. We offered 8 weeks of outdoor PYSP sessions from August–October 2021. The program was offered from 6 pm–7 pm 3 days per week (Monday, Wednesday, and Friday). The outdoor grass playing area was demarcated using plastic cones and was 100' × 50' (i.e., approximately the size of a regulation basketball court). This size was chosen deliberately to bring players closer to one another, allowing more ‘touches’ during games, and to increase the feasibility of conducting indoor sessions in subsequent versions of the program. Because we offered the program during the COVID-19 pandemic, we implemented several safety precautions suggested by amateur and professional sport agencies and required by the university (e.g., pre-participation health screenings, temperature checks upon arrival). However, mask wearing was not required.

The sport game played changed at the beginning of each week, rotating between Ultimate Frisbee, Ultimate football, handball, netball, and soccer (see Table 3 for full game rules and equipment). Each session began with a 5-min warm-up period of ‘catch’ between participants using the sport implement of the day (e.g., ball, Frisbee). Following the warm-up period, the leader reviewed the rules of each game and selected teams randomly. Because the participants were sedentary and nearly 50% had never participated in an organized sport, play periods during the first 2 weeks were 6-min long (i.e., 30-min of total playing time) followed by a 5-min hydration and socialization break, to allow adaptation to increased PA. During the remaining 6 weeks, each period was 8 min (i.e., 40 min of total playing time) followed by 3-min breaks. The leader also played, switching teams after each period. See Table 3 for a more detailed explanation of PYSP rules and equipment.

**Table 3:** PYSP general rules, specific game rules, and equipment

General PlayFit game rules	
• Players will focus on maintaining arms-length distance from other players to avoid contact (i.e., follow the personal 'bubble rule') • Players may attempt to pass or score from any location (i.e., no offside rules, etc.) • Players may hold ball for up to 3 s before attempting to pass or score • Out of bounds passes/shots are turned over to the other team	
Specific sport game rules	Equipment
Soccer	• 100' × 50' Field boundary markers (e.g., cones) • Molten™ lightweight volleyball inflated to approximately 2.0 pounds per square inch (PSI) • Two (2) 4' × 6' collapsible goals
Ultimate football	• 100' × 50' Field and end zone boundary markers • Nerf™ foam football
Handball	• 100' × 50' Field boundary markers • 5" rubber dodgeball at approximately 1.8 PSI • Two (2) 4' × 6' collapsible goals
Ultimate Frisbee	• 100' × 50' Field boundary markers • ChuckIt! Zipflight™ foam Frisbee
Netball	• 100' × 50' Field boundary markers • Two (2) 8ft portable or permanent basketball hoops • Youth basketball (size 5) at approximately 8.0 PSI

### Measures

#### Program attendance and adverse events

The PYSP leader used a REDCap [60] survey to record attendance at each session, weather conditions at the site, and report any adverse events, while participants could report them via a monthly survey or by contacting research staff.

#### Adolescent acceptability questionnaires

Program satisfaction was measured at week 8 using several face-valid items. The first item asked, ‘Would you recommend PlayFit to a friend?’ (Yes/No). If yes, participants were presented with a ‘Net Promoter Score’ item [61] asking how likely they were to recommend the PlayFit program to a friend, using a scale of 0 (‘Not at all likely’) to 10 (‘Extremely likely’). The next item asked, ‘Do you look forward to doing PlayFit?’ (Yes/No). If yes, participants responded to the following question ‘I look forward to doing PlayFit…’ with the options being 1 (‘Not very much’), 2 (‘A little’), and 3 (‘Very much’). Participants rated the next item ‘How would you feel if you could no longer do PlayFit?’ using the options 1 (‘Very disappointed’), 2 (‘Somewhat disappointed’), or 3 (‘Not disappointed’). The participants next provided their rating of the program leader from 1 (‘Poor’) to 5 (‘Excellent’). The participants could also explain their ratings of each of the items qualitatively.

#### Individual sport game ratings

At week 8, the adolescents rated their level of satisfaction with each of the 5 sport games using the scale 1 ‘(I did not like it’), 2 (‘I liked it a little’), or 3 (‘I liked it a lot’). The participants could also explain their ratings of each of the items qualitatively if they answered with a rating of 1 (i.e., ‘I did not like it’).

#### Guardian acceptability questionnaire

The guardians of the participants completed 6 open-ended items at week 8. The included items asked (1) what they may enroll their child in as an alternative to the PYSP, (2) what they thought the main benefit of the PYSP was for their child, (3) what aspects we should not change, (4) whether they would recommend the PYSP to a friend, (5) whether they would enroll their child if the PYSP were offered in the future, and (6) suggestions for how we could improve future iterations of the PYSP.

#### Goal orientation

The 13-item Task and Ego Orientation in Sport Questionnaire (TEOSQ; [47] was used to measure goal orientation at baseline. Each item was prefaced by the stem, ‘I feel most successful in sport when…’ Task-orientation items include examples such as ‘I learn a new skill and it makes me want to practice more, ‘while examples of ego-orientation items include 'I'm the only one who can do the play or skill.’ Each item is rated using a 5-point Likert-type scale. The reliability of each scale was *good* to *excellent* (i.e., 0.86 for the task scale and 0.85 for the ego scale).

#### Overall physical activity enjoyment

Enjoyment of physical activity was measured using the Short Physical Activity Enjoyment Scale (S-PACES; [62], developed for children in grades 3–6. The S-PACES consists of 7-items rated using 5-point Likert-type scales. Each item begins with the stem, 'When I am active…' with sample endings including ‘I feel bored’ and ‘I dislike it.’ The reliability of the scale was *good* (0.85).

#### PlayFit Youth Sport Program enjoyment

Enjoyment of the PYSP was measured at week-8 using a modified version of the S-PACES. The original stem ‘When I am active…’ was replaced with 'When I do PlayFit…' followed by the endings of the original scale and using the same 5-point Likert-type scales. The reliability of the scale was *excellent* (0.97).

#### Situational needs satisfaction

At week 8, the 12-item Activity Feelings State Scale (AFS; [63] was used to measure whether the PYSP satisfied the basic psychological needs of competence, autonomy, and relatedness. The participants responded to the stem 'Participating in PlayFit made me feel …' followed by such items as ‘free to decide for myself what to do’ (Autonomy scale), 'like my skills are improving’ (Competence scale), or ‘involved with close friends’ (Relatedness scale). The participants rated each item on a 7-point Likert-type scale. The reliability of the scales was *questionable* to *good* (i.e., 0.65 for autonomy, 0.88 for competence, and 0.50 for relatedness).

#### Accelerometry

Each participant wore ActiGraph (Pensacola, FL) wGT3X-BT tri-axial accelerometers around the waist for 7 days prior to the sport game sessions to measure baseline physical activity. Intra-session accelerometry data for Ultimate Frisbee and Netball were improperly recorded and unusable. As recommended by Trost and colleagues [64], time spent sedentary and in physical activity was determined using the cutoffs proposed by Evenson and colleagues (i.e., sedentary ≤ 100, light 101–2295, moderate ≥ 2296, and vigorous intensity ≥ 4012 counts per minute; [65]. To reflect the intermittency of the games of PlayFit, the accelerometers recorded at 3-s epochs.

### Data analysis

#### Quantitative data

Demographic data are presented as medians and/or counts. Because of the small sample size, scores for the outcome variables are reported as medians and ranges, total out of total number possible, or percentages of the total possible.

#### Qualitative data

Qualitative data were analyzed by two researchers independently using deductive (i.e., top-down) and inductive (i.e., bottom-up) content analysis to categorize the responses into specific themes. Deductive content analysis was used a priori to generate broad themes. Inductive analysis was used to allow additional themes not predicted by the researchers a priori to emerge. Once the themes were established, the frequencies for each were tabulated.

## Results

### Objective 1. Recruitment capability and resulting sample characteristics

**Fig. 1:**
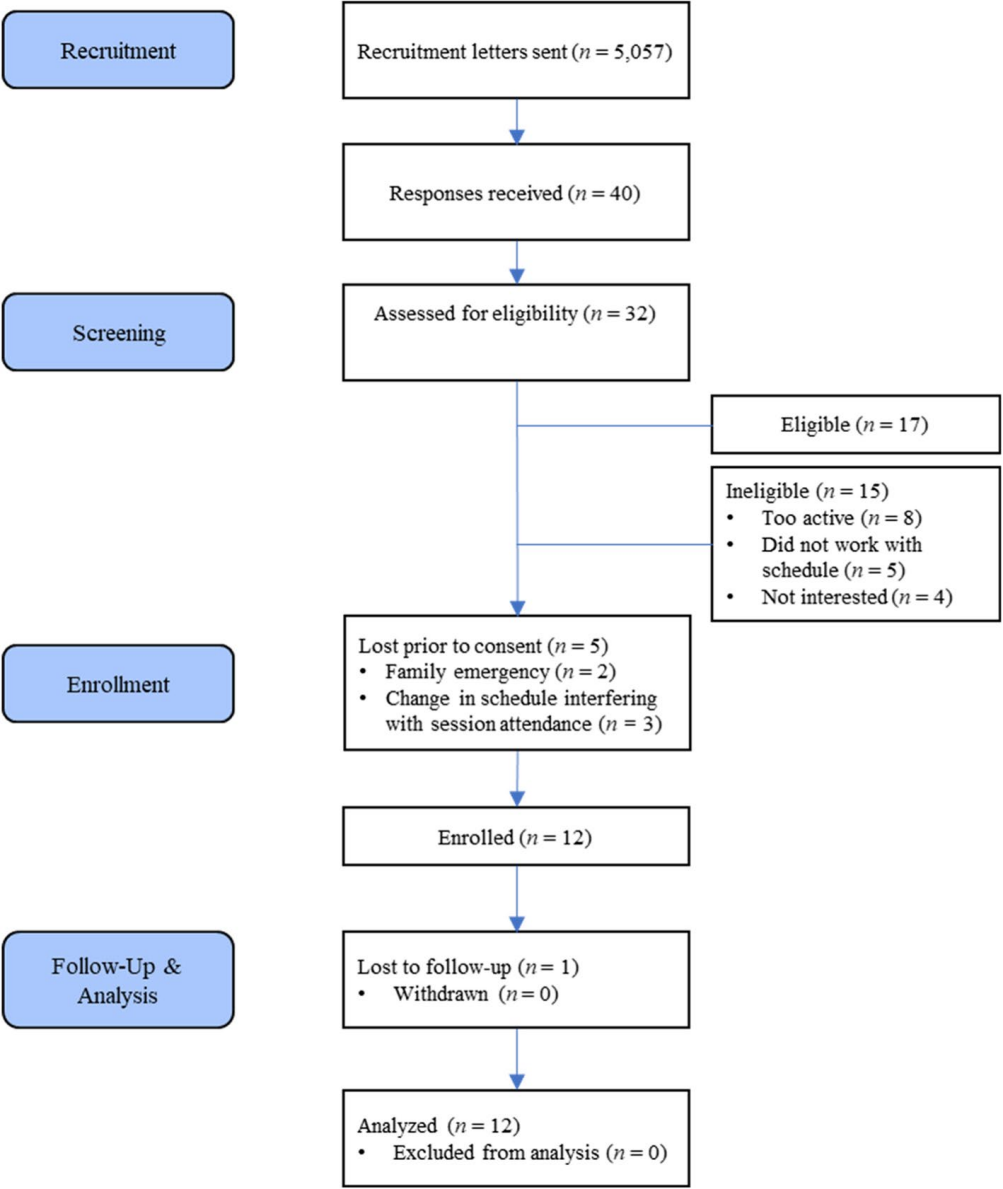
Participant flow throughout the intervention

Twelve White adolescents 10 to 12 years of age and in grades 5 to 7 enrolled and consented (baseline data are illustrated on Table 4). The participants were of mostly ‘healthy weight’ according to the Centers for Disease Control and Prevention (i.e., between the 5th and 85th percentile for their age and sex; [61]). Seven of 12 participants reported playing an organized sport in the past and 3/7 were doing so currently. One 11-year-old male participant was lost to contact following baseline measurements (due to reasons not provided), leaving 11 participants to complete the program (see Fig. 1 for participant flowchart).

**Table 4:** Participant baseline characteristics

		*n* (%)
Sex	Female	7 (58.3%)
	Male	5 (41.7%)
Age	10	2 (16.6%)
	11	6 (50.0%)
	12	4 (33.3%)
Grade	5	3 (25.0%)
	6	6 (50.0%)
	7	3 (25.0%)
Race or ethnicity	White	12 (100.0%)
Sport experience	Played organized sport in the past	7 (58.0%)
	Currently played an organized sport	3 (25.0%)

#### How many potential eligible members of the targeted population are accessible in the local community?

We mailed 5,057 recruitment letters using a listing of pediatric healthcare patients. This system allowed us to partially prescreen participants by general characteristics, including age, but not by other eligibility requirements (e.g., physical activity level). There was no other direct public involvement or patient involvement through the College of Medicine, besides accessing pediatric patient databases to mail recruitment letters.

#### What are the recruitment rates?

Forty guardians responded with interest in enrolling their children and 32 continued to correspond to be assessed for eligibility. Seventeen were deemed eligible for the study and 5 were lost prior to consent, leaving 12 participants to consent and enroll in the study.

#### How feasible and suitable are eligibility criteria?

The eligibility criteria did not appear to be overly restrictive or too inclusive. The biggest challenge to recruitment may have been related to COVID-19 restrictions, as explained below.

#### What are the obstacles to recruitment?

The most meaningful obstacle of this study was delays to beginning human-subjects research related to COVID-19 mandates. These delays forced an abbreviated recruitment schedule to allow the outdoor intervention to be held in its entirety prior to the early darkness and lower temperatures of the fall. Therefore, letters were mailed between July 19 2021 and August 06 2021 for an intervention start date of August 9 2021 and an end date of October 10 2021. At the time, approximately 2000–4000 new COVID-19 cases were reported daily in Pennsylvania [66]. Although well-ventilated outdoor settings are associated with reduced risk of infection [67, 68], perceptions that participation may have been unsafe could have limited recruitment. Other important obstacles were that bedtimes, homework, and other evening activities conflicted with the ability of children to attend.

#### How relevant is the intervention to the intended population?

The participants in the study showed need for the intervention, as to qualify, their daily physical activity was below the minimum recommendation of 60-min per day of MVPA [69]. The median accelerometer-derived MVPA of the participants at baseline was 28.5 min/day (range = 4.2–41.1). In addition, the guardians of the participants expressed a desire to enroll their child in a physical activity program that their children would like and adhere to.

#### Are the characteristics of the study participants consistent with the range of expected characteristics as informed by the research literature?

The characteristics of the participants were mostly consistent with what was predicted based on the theoretical design of the PYSP. For example, the participants typically had higher scores for task-related compared to ego-related goal-orientation items. Moreover, their median physical activity levels at baseline were below the eligibility criteria of no more than 60 min/day of MVPA.

### Objective 2. Evaluation and refinement of data collection procedures and outcome measures

#### How feasible and suitable are the data collection procedures?

There were no issues provided by the adolescents or guardians in understanding the questionnaires and data collection instruments. Possibly due to attention checks (e.g., 'a question was not answered. Would you like to answer this item?'), every questionnaire that was started was completed.

#### How feasible and suitable is the amount of data collection?

No participants reported feeling overwhelmed or burdened by the amount of data collection at each time point, with most spending fewer than 15 min completing the items.

#### Do the measures appear to be performing in a consistent way with the intended population as compared to measurement information available in the research literature?

The reliability coefficients of the questionnaires were ‘good’ to ‘excellent,’ except for the ‘autonomy’ and ‘relatedness’ subscales of the Activity Feelings State Scale. Due to the small sample size, outliers could have biased these estimates. 

### Objective 3. Acceptability and suitability of intervention and study procedures

#### What are the retention and follow-up rates as the participants move through the study and intervention?

Eleven of the 12 (91.6%) participants who consented and enrolled at baseline remained in the study at the end of the 8-week intervention.

#### What are the adherence rates to study procedures, intervention attendance, and engagement?

The participants attended a median of 12 (range = 6–13) of 16 sessions offered. While 24 sessions were originally planned, 6 were canceled due to severe weather (e.g., lightning and/or heavy rain) and 2 by leader absence. Besides cancelations, the most common reasons for missed sessions were conflicting commitments such as band practice, other extracurriculars, interference with bedtimes or schoolwork, and/or lack of transportation.

Every assigned questionnaire was completed by 12/12 enrolled adolescents at baseline and 10/11 at week 8. The single adolescent that did not complete every questionnaire at week 8 completed 4/5 questionnaires. Additionally, 12/12 guardians at baseline and 11/11 at week 8 completed every questionnaire.

#### What is the level of safety of the procedures in the intervention?

The participants and guardians reported no adverse events during or following the intervention.

### Objective 4. Resources and ability to manage and implement the study and intervention

#### Does the research team have the administrative capacity, expertise, skills, space, and time to conduct the study and intervention?

The intervention sessions were delivered by the lead author. Recruitment and enrollment activities were carried out by one research project manager and one research technologist. The administrative tasks of the current intervention could likely be achieved using fewer personnel.

#### Can we conduct the study procedures and intervention in an ethical manner?

The present study complied with all approved human-subjects’ protocols as well as federal, state, and University COVID-19 pandemic mandates. There were no adverse events reported throughout the intervention period by the adolescents or their guardians, but adverse advent reporting was to be completed by the PYPS leader or reported to research project staff.

#### Can the study and intervention be conducted within the designated budget?

The budget for the present study was not precisely estimated prior to the intervention*.* However, the intervention was delivered below the budget submitted during the funding application.

#### Is the technology and equipment sufficient to conduct the study and intervention, including collection, management, and analysis of data?

REDCap longitudinal data collection software was sufficient to conduct the study and was available through contracts with the University. Basic data entry skills and training are necessary to input these data accurately. Depending on the sample (e.g., access to the internet), paper format instruments are available if the need for a non-electronic measurement delivery is deemed necessary.

#### Are we able to efficiently and effectively manage data entry and analysis?

REDCap offered the study team members a seamless and efficient means to manage data collection.

### Objective 5. Preliminary evaluation of participant responses to intervention

#### Does examination of quantitative data suggest that the intervention is likely to be successful?

The quantitative data suggest that, in its current form, the intervention could be a promising approach to promote activity among sedentary adolescents. Following the conclusion of the program, 8/10 participants who completed all post-study satisfaction items reported that they would recommend the PYSP to one of their friends, and their median net promoter score (i.e., likelihood of recommending the program to a friend, out of 10) was 7 (range = 6–10). Nine of 10 reported looking forward to the sessions with a median rating (out of 3) of 2.5 (range = 1–3). The median ratings (out of 3) for the individual sport games ranged from 3 (range = 2–3) for handball and Ultimate frisbee (range = 2–3), to 2 for soccer (range = 1–3), Ultimate football (range = 2–3), and netball (range = 1 – 3). Finally, 8/10 participants responded that they would be interested in continuing the program if it were offered to them in the future. Individual counts and proportions for each item are included in Table 5.

**Table 5:** Participant Net Promoter Scores, satisfaction with the leader, and satisfaction with the individual games of the PYSP

Variable	Rating	*n* (%)
Net Promoter Scores	6	3 (42.8%)
	7	1 (14.2%)
	8	1 (14.2%)
	10	2 (32.6%)
Leader ratings	3 (Good)	1 (10.0%)
	4 (Very good)	4 (40.0%)
	5 (Excellent)	5 (50.0%)
Handball ratings	2 (I liked it a little)	3 (30.0%)
	3 (I liked it a lot)	7 (70.0%)
Soccer ratings	1 (I did not like it at all)	1 (10.0%)
	2 (I liked it a little)	4 (40.0%)
	3 (I liked it a lot)	5 (50.0%)
Ultimate Frisbee ratings	2 (I liked it a little)	4 (40.0%)
	3 (I liked it a lot)	6 (60.0%)
Ultimate football ratings	2 (I liked it a little)	5 (50.0%)
	3 (I liked it a lot)	5 (50.0%)
Netball ratings	1 (I did not like it at all)	2 (20.0%)
	2 (I liked it a little)	4 (40.0%)
	3 (I liked it a lot)	4 (40.0%)

In general, scores for *overall* physical activity enjoyment (median = 23.0, range = 22–35) were lower than enjoyment of the PYSP (median = 26.5, range = 21–35). However, we were not adequately powered to examine these differences statistically.

Among the 3 sport games evaluated for MVPA, the proportion of total playing time spent in MVPA was between 30–50% and 68–91% for all physical activity intensities. That is, despite providing little direct encouragement beyond the self-referenced ‘try as hard as you can,’ the proportion of time in MVPA was comparable to more traditional sport games (e.g., 48.3% ± 13.9%; [70] and practices (e.g., ~ 33%; [71, 72].

Finally, at week 8, 10/11 guardians reported that they would consider enrolling their children in the program if it were offered in the future.

#### Do participants or relevant others provide qualitative feedback that may be indicative of the likelihood that the intervention will be successful?

The qualitative responses provided useful information that could be used to improve future versions of the PlayFit program. For example, the participants noted the characteristics that they liked most about the leader, who, based on the ‘positive coaching’ dimension of the fun-integration theory, was theorized to play an integral role in facilitating experiences of fun during program sessions. The participants reportedly liked that the leader was encouraging, supportive, fair, and tried to include everyone (i.e., focused on providing every player ‘touches’ during games). The responses also suggested that the games of PlayFit were easy to play with few skill barriers to entry, were low pressure (i.e., did not introduce pressure to score or to perform), provided an opportunity to sample a variety of sport games, and a chance for socializing with similar peers. However, changes to netball and Ultimate football were suggested. For netball, the participants indicated the regulation size basketball hoop was too difficult to score on, while the foam ball for Ultimate football was easy to catch but no easier to throw than a normal football (qualitative responses among the adolescent participants are summarized in Table 6).

**Table 6:** Adolescent participant responses to program satisfaction qualitative items at week 8

Item	Theme	*n*
Why did you feel this way (good or bad) about your PlayFit leader?	• The leader was kind/nice	5
	• The leader was funny	1
	• The leader made program fun/enjoyable	2
	• The leader demonstrated fairness	2
	• The leader demonstrated inclusivity	2
	• The leader provided clear instructions	2
	• The leader was a motivator	1
	• The leader was helpful	1
What do you LIKE about PlayFit?	• Liked the variety of sport games	3
	• Liked ‘everything’ about the program	2
	• Liked that the sport games were fun/enjoyable	2
	• Liked ‘almost everything’ about the program	1
	• Liked the socialization with peers	3
	• Like that there was no pressure to perform/score	1
	• Liked to challenge oneself	1
What do you NOT LIKE about PlayFit?	• ‘Nothing’ was not liked	4
	• Did not like soccer	1
	• Scheduling conflicts with homework	1
	• Heat/Humidity	1
	• Inclement weather cancelations	1
	• Spotted Lanternflies in playing field	1

Note that *n* represents the total number of mentions of the lower-order theme. Some participants provided multiple lower-order themes in a single response

The guardians also provided helpful qualitative feedback. Interestingly, several guardians reported not knowing what other sport programming would be satisfactory for their children, highlighting the unique needs of this group. Most reported encouraging their children to participate in individual pursuits (e.g., Karate) or walking. Some opined that increasing competition and specialization made it difficult for their child to wish to participate in youth sport. Many guardians responded that what they liked about the program was that their children were exercising and socializing with other children, trying a multi-activity program, and that it was a non-competitive environment with little pressure to perform (qualitative responses from the guardians of the participants are summarized in Table 7).

**Table 7:** Guardian responses to program satisfaction qualitative items at week 8

Item	Theme	*n*
What is the main benefit you feel your children get from PlayFit?	• Provided more physical activity/exercise	9
	• Provided more time outdoors	3
	• Was a sedentary behavior (e.g., internet) alternative	1
	• That it was a safe program	1
	• Provided opportunities for socialization with peers	9
	• Provided opportunities to play with others	6
	• Provided a variety of sport games	2
	• Fostered cooperation and leadership	2
	• Fostered a non-competitive environment	1
How can we improve PlayFit to better meet the needs of you and your children?	• Have alternative location for inclement weather	4
	• Offer more sessions at alternative times	2
	• Offer fewer sessions	1
	• Enroll more children to make up for those who may be absent	1
	• Would not change anything	1
	• More time getting to know one another at the beginning	1
What should we not change? What part of this program worked so well that we should leave it as is?	• Keep the variety of sport games	3
	• The lack of resemblance to other team sports, such as not using a whistle, or expecting kids to score or throw properly	3
	• The simplicity to help kids that are not as athletically inclined	1
	• Continue to allow kids to each choose how hard they would like to work	1
	• The option to attend different days	1
	• One-hour sessions work well	1
	• Communication with parents/guardians was adequate	1

Note that *n* represents the total number of mentions of the lower-order theme. Some respondents provided multiple lower-order themes in a single response

#### Are the findings congruent with the proposed theoretical model for the intervention?

The qualitative and quantitative data collected during this small-scale feasibility study could offer preliminary support for the theoretical model proposed in the design section of this manuscript.

## Discussion

### Strengths

Retention among those who enrolled in the intervention was high (i.e., 91.7%), providing support for its feasibility. Although several sessions were canceled due to leader absence or  inclement weather, those that were not canceled were delivered in their entirety. The responses, both quantitative and qualitative, among the adolescents and their guardians suggested that the program required only minimal modification to improve the experience of the stakeholders.

### Challenges

Initial recruitment was hampered by an abbreviated timeline and the impact of the COVID-19 pandemic. Many guardians and adolescents may have been fearful of enrolling in a program where close contact would occur. Other activities, such as other extracurriculars, homework, and scheduled bedtimes also impacted session attendance. Moreover, several sessions were canceled due to inclement weather or unexpected leader absence, which limited the total sessions available to the participants.

### Recommended refinements

Despite the short recruitment timeline, the manner in which the study was advertised may have limited uptake. Participants were recruited via physical mail which may not have been checked on a regular basis. Although the letters appeared to be from the medical provider of the child, they could have been misidentified as 'junk' mail and discarded. Though physical mailings may be a component of an effective recruitment campaign, in the future we could incorporate more “word of mouth” approaches to garner interest. For example, a more outward focus on the strengths of the program relative to existing sport options for adolescents (e.g., non-competitive/contact, easy to play at any skill level, no pressure to perform) may help increase recruitment. In addition, social media advertising and video media “commercials” demonstrating what a session of the PYSP looks like, in practice, may prove attractive.

Some of the responses from the adolescents also suggested advertisement might be more successful if there were more focus on its atypical, unstructured, and non-competitive design. For example, the 3-s rule did not allow players to hold the sport implement (e.g., Frisbee, soccer ball) for extended periods. Along with mirroring the movement patterns of children and allowing them to self-regulate their effort, the intention was to involve more players by preventing more skilled players from ‘hogging the ball’ or running from ‘end-to-end’ to score. However, we understand that this could be frustrating for more skillful or ego-oriented players.

Based on the observations of the PYSP leader and the qualitative responses of the participants, there were a few minor adjustments to the sport games of the program that may improve the experience. For example, the ball used for ultimate Football (a foam football) was easy to catch but difficult to throw. In future iterations, the use of a football similar to the Nerf™ Vortex (i.e., with a tail for stabilization) may prove easier to use for Ultimate football. Moreover, a larger sized net may allow for more scoring opportunities for lower-skilled players.

### Limitations

Despite the encouraging results, several important limitations should be considered. First, because this was a formative evaluation of a preliminary version of the PYSP, we did not have formal feasibility hypotheses, nor prespecified feasibility thresholds or progression criteria. In addition, current methodological limitations precluded us from empirically evaluating experiences of fun *during* sport without interrupting gameplay. Therefore, we cannot conclude whether retrospective perceived sport enjoyment of the participants was influenced by experiences of fun during the PYSP sessions, or other unmeasured variables. Future studies utilizing noninvasive, measures of automatic affective (i.e., fun) responses during sport, such as facial expressions [73] or other psychobiological responses [74], may help to elucidate the mechanisms underlying differences in perceived enjoyment.

Generalizing these findings is difficult due to the small, homogeneous sample and short intervention duration. The participants were all White, higher socioeconomic status (mean family income = $125,000), and had regular transportation to the evening sessions. Children whose guardians worked multiple jobs or abnormal schedules may not have had similar access. We also conducted this study from August to October 2021, during the COVID-19 pandemic. Adolescents who expressed interest and guardians willing to enroll their children may have been unique in that they felt safe with proximity to others in the throes of the pandemic. Finally, at this point in the development of the PYSP, we did not conduct an economic evaluation.

## Conclusions

In its current form, the PYSP shows signs of promise. However, before continuing to an appropriately-powered efficacy trial, it should be further refined based on the present recommendations. We anticipate that some criticisms of the PYSP will center on the contention thatoutcomes such as increased fitness, skillfulness, and performance should be considered ‘secondary’ relative to maximizing repeated experiences of fun during sport. We understand this criticism but suggest that improvements in these secondary outcomes may follow naturally when children discover a sport (or PA) to which they adhere. That is, to reap the benefits of PA, people must adhere to PA.

Moreover, because the PYSP is intended to be an alternative to typical elite and recreational sport, we do not explicitly aim to advance players to the ‘next level.’ Therefore, changes in fitness, skillfulness, and performance matter less and participants can self-reference them (if they wish to do so, at all). For example, participants in the PYSP may freely decide to remain in the program indefinitely, try (or return to) an elite or recreational sport, or drop out altogether to pursue other, more enjoyable, physical activities. Therefore, in contrast to many athlete-development models, the intention of PYSP-style programming is to increase the likelihood of *lifelong* PA among those whose needs are not being met by existing sport programs. The PYSP may also help rectify one of the common shortcomings of recreational sport programs—specifically that their quality depends on the mission of the program as well as the personality types, experiences, and expertise of those leading them [38, 40–42]. That is, children who participate in the PYSP may one day become adults who can more easily empathize with, and lead, children like themselves as children (i.e., those who experienced typical youth sport as not fun). As with how many elite and recreational sport participants return to coaching as adults, the PYSP could generate a ‘grassroots’ network of future leaders, helping to disseminate the program and insure its long-term survival. 

## Supplementary Information

**Additional file 1. **CONSORT 2010 checklist of information to include when reporting a pilot or feasibility trial*.

## Data Availability

N/A.
